# Barriers to healthcare and a ‘triple empathy problem’ may lead to adverse outcomes for autistic adults: A qualitative study

**DOI:** 10.1177/13623613231205629

**Published:** 2023-10-17

**Authors:** Sebastian CK Shaw, Laura Carravallah, Mona Johnson, Jane O’Sullivan, Nicholas Chown, Stuart Neilson, Mary Doherty

**Affiliations:** 1 Brighton and Sussex Medical School, UK; 2 Michigan State University, USA; 3 NHS Digital, UK; 4 Cork University Hospital, Ireland; 5 London South Bank University, UK; 6 Independent Researcher, Ireland

**Keywords:** adults, autism, autistic, epistemic injustice, healthcare, health services, insider research, minority stress theory, qualitative research, triple empathy problem

## Abstract

Autistic people experience more co-occurring health conditions and, on average, die younger than non-autistic people. Despite growing awareness of health inequities, autistic people still report barriers to accessing healthcare. We aimed to explore the experiences of autistic people accessing healthcare, shining a light on the complex interplay of relevant factors and to explain, at least in part, the possible reasons underling health disparities and adverse health outcomes. This is a qualitative study from an autistic research team. Data were collected from 1248 autistic adults as part of a large, mixed-methods, international survey exploring barriers to primary healthcare. This article reports the qualitative findings, following a thematic analysis. Using our exploratory findings, we then constructed a model to explain the reported experiences. Respondents reported a variety of barriers. Here, our article gives voice to their stories, in their own words. Themes included: early barriers; communication mismatch; doubt – in oneself and from doctors; helplessness and fear; and healthcare avoidance and serious adverse health outcomes. Our constructed model outlines a chronological journey through which healthcare access barriers may lead to adverse health outcomes. Our findings also build on the double empathy problem, situating this in a medical context, proposing a **triple empathy problem**.

**Lay abstract:**

Autistic people live with more mental and physical health conditions and, on average, die younger than non-autistic people. Despite widespread commitments to tackling these issues, autistic people still report various barriers to accessing healthcare. This article aims to explore the area in depth, from the perspective of autistic people. This research benefits from being led by autistic people, for autistic people – all of the researchers are autistic, and most of us are also medical doctors. Data, in the form of written comments and stories, were collected as part of a large survey. Here, we explored these for common themes and possible deeper meaning within the experiences. People who took part reported a variety of barriers. Here, our article gives voice to their stories, in their own words. Themes included: early barriers; communication mismatch; doubt – in oneself and from doctors; helplessness and fear; and healthcare avoidance and adverse health outcomes. Our findings allowed us to create a model that aimed to understand and explain the reported barriers in the context of the previously known consequences. We also built on wider autism theories to explain our findings in more depth.

## Introduction

Autistic people experience and interact with the world differently compared to non-autistic people. Autism is associated with a wide variety of lifelong communication, behaviour and sensory differences (Royal College of Psychiatrists, 2020). While most clinical research has traditionally focused on autistic children, the majority of autistic people are in fact adults (Royal College of Psychiatrists, 2020). Despite being key gatekeepers to formal diagnosis, and although there is increasing identification of autistic adults in general, doctors are known to underestimate the amount of autistic people within their patient population (Zerbo et al., 2015). Due to prevailing stereotypes and a historical tradition of deficit-focused approaches to screening and diagnosis, the majority of autistic adults are indeed likely to be undiagnosed, particularly those without co-occurring intellectual disability (Brugha et al., 2011). Such stereotypes may be perpetuated through an epistemic injustice, whereby autistic people may not be seen as credible sources of information in the evolving understanding of autism in medical contexts. Epistemic injustice can be defined as ‘harms that relate specifically to our status as epistemic agents, whereby our status as knowers, interpreters, and providers of information, is unduly diminished or stifled in a way that undermines the agent’s agency and dignity’ (Chapman & Carel, 2022). Centring autistic voices within the evolving research discourse around autistic healthcare may, therefore, be key to producing an ethically sound evidence base that advances social justice and health equity for autistic people (Kidd et al., 2023).

Realising that you are autistic during adulthood can be a life-affirming experience, following years of conformity to societal expectations and feelings of unvalidated difference (Stagg, 2019). Achieving this realisation (with or without formal diagnosis) can allow one to begin processing decades of internalised ableism and to flourish as their true self (Nguyen et al., 2020). Living in a non-autistic world can be highly stressful for autistic people, particularly prior to discovering one’s own autistic identity (Lilley et al., 2022). The impact of chronic stress on wellbeing and mental health is clear. Minority stress theory explains how negative life experiences – such as prejudice, rejection or having to hide one’s true identity or mask, for example – can feed into poor mental and physical health, through the cumulative impact of chronic and acute stressors (Botha & Frost, 2020; Lick et al., 2013). Autistic people are often subject to traumatic experiences and discrimination within current socio-political and environmental expectations/settings (Griffiths et al., 2019). Such experiences undoubtedly take their toll on wellbeing. In keeping with minority stress theory, autistic people also live with poorer physical and mental health, more co-occurring conditions and on average die younger than non-autistic people (Doherty et al., 2022; Hirvikoski et al., 2016; Royal College of Psychiatrists, 2020; Wallen et al., 2018). The United Kingdom 2021 National Autism Strategy acknowledged the 16-year mean life expectancy reduction for autistic people (HM Government, 2021) demonstrated by Hirvikoski et al (Hirvikoski et al., 2016). Despite this, autistic adults report a litany of barriers to having their healthcare needs met, starting right from the point of initial access (Doherty et al., 2022). We are more likely to need to use emergency room services, more likely to be admitted when using them and more likely to die during these admissions than non-autistic people (Vohra et al., 2016). In line with the UK National Health Service’s (NHS) constitutional core value that ‘Everyone Counts’ (Department of Health and Social Care, 2021), the UK Government has committed to closing the life expectancy gap, to ensure autistic people ‘lead full, and happy lives’ (HM Government, 2021). Central to this ambition is tackling the health inequities experienced by autistic people (HM Government, 2021). This may be achieved through improved understanding by health and care professionals, and through improving other contributory factors, previously reported, such as late presentation of serious illness or under-acknowledged clinical signs by professionals (Doherty et al., 2021; Haydon et al., 2021).

Multiple studies have described the barriers to healthcare for autistic people (Mason et al., 2019; Vogan et al., 2017). A recent study of our own also found similar access barriers, and more importantly, correlations with self-reported adverse health outcomes (Doherty et al., 2022). In this cohort, 80% of autistic respondents reported difficulty accessing a General Practitioner (GP) when required and, most worryingly, over one third of autistic respondents reported not seeking medical help for ‘potentially serious or life-threatening’ conditions (Doherty et al., 2022). Several qualitative studies have explored experiences of accessing healthcare from the perspectives of autistic people, their carers or supporters and clinicians (Calleja et al., 2022; Dern & Sappok, 2016; Mason et al., 2021; Nicolaidis et al., 2015). Factors impacting healthcare access are described at system-level, provider-level and individual-level, which mirror issues faced by autistic people when accessing other services such as education (Anderson et al., 2018). However, the impact of autistic accounts of healthcare access have yet to result in widespread service improvements. Efforts to improve healthcare access for autistic people require a deep understanding of the factors leading to and the consequences of inaccessible healthcare from the perspective of autistic people. Meaningful understanding of these links from an autistic perspective is, however, lacking in the current literature. Therein lies the importance of this study.

Within this article, we analyse and report the qualitative findings from an international, cross-sectional survey. Our quantitative findings have already been published elsewhere (Doherty et al., 2022). Our primary aim here was to explore the experiences of autistic adults in relation to healthcare access barriers and self-reported adverse outcomes. As a secondary aim, we hoped to scrutinise the available data for potential deeper meaning, drawing on our insider status, to better understand how healthcare experiences may *lead* to adverse outcomes for autistic people. No prior studies have focussed on understanding the meaning attributed to experiencing access barriers, nor the ways in which such barriers may be linked to adverse health consequences.

## Methods

### Participants and data collection

This study is based on pre-existing, previously unanalysed data. This article reports data relating to autistic adult respondents. Both formal diagnosis and self-identity were acceptable. Data were collected using online surveys that explored barriers to primary healthcare and adverse health outcomes for autistic people. The main focus was on experiences of General Practice and with GPs. The final survey itself, including detail on its construction and piloting, is published elsewhere as a quantitative study (Doherty et al., 2022). This survey recruited participants via social media and an autistic charity website (AsIAm) using a convenience sampling approach (Doherty et al., 2022). Within this article, we analyse and report the qualitative responses from both the pilot and main surveys. The qualitative options did not change between these two phases of the project, and thus we chose to combine them. Our data were gathered in 2018 and 2019. Table 1 outlines questions with the option for free-form responses, and the number of such responses received.

**Table 1.** Questions receiving qualitative responses included for analysis.

Survey question	Number of qualitative responses
Why do you usually visit your doctor?	*N* = 85
Which of the following communication issues cause you problems during a consultation?	*N* = 160
Which communication issue causes you the MOST problems during a consultation?	*N* = 49
Do you experience sensory issues which make it difficult to visit your doctor?	*N* = 129
What communication methods do you use?	*N* = 36
What communication methods do you AVOID if possible?	*N* = 22
If your GP offered options for making an appointment, which would you be most likely to use?	*N* = 30
Visits to my doctor would be easier if	*N* = 130
Have you ever had a mental health condition remain untreated due to difficulties accessing healthcare?	*N* = 22
Have you ever had a physical health condition remain untreated due to difficulties accessing healthcare?	*N* = 18
If you answered yes to any of the last 6 questions, would you like to give more details?	*N* = 548
Please give any further information or suggestions here	*N* = 297

GP: General Practitioner.

### Data analysis

Data were analysed by SCKS and MD over two phases. First, using reflexive thematic analysis, following the approach of Braun and Clarke (2006). SCKS and MD immersed themselves in the data. This was achieved through reading and re-reading. Initial coding was undertaken by a single researcher (SCKS). This was done using the Microsoft Word comments feature. This was verified by MD. SCKS and MD then came together to jointly search for, review and define themes. This was an iterative process, which concluded when both researchers agreed on the final, constructed analysis.

Following this thematic analysis, SCKS and MD stepped beyond this to construct a model that sought to explain the potential links between healthcare experiences and adverse outcomes for autistic people. This approach was grounded in their own insider status as autistic people, as medical doctors, and through having conducted the initial thematic analysis. This was an iterative process where the resulting model was increasingly detailed and refined over numerous versions until both were happy that this reflected their subjective interpretations of the data in the context of their own insider positioning. This innovative process embraced subjectivity and embodied a co-constitutional approach to the generation of this new knowledge (Flood, 2010). In short, our own experiences were used to provide deeper insight into the reported data, in the search for hidden meaning, embracing a relativist ontology. Similarly, this approached epistemological assumptions from a social constructionist perspective (Andrews, 2012). Social constructionism proposes that collective human knowledge is created through social interaction (Harris, 2006). This embraces the subjective nature of individual experiences, beliefs and practices, embedded in our own constructed knowledge (Shaw, 2021). Taking this stance allowed us to consider both chronology and possible underlying causation more deeply, resulting in the graphical representation of a model.

### Ethical approval

This study was approved by the SJH/TUH Research Ethics Committee at Tallaght University Hospital.

### Insider positioning/community involvement

This study benefits from an insider approach. Our research team includes autistic individuals from a variety of disciplines. Most of us are medical doctors. This insider perspective was vital to the design and undertaking of this study. As Chown et al. explain, ‘it is both epistemologically, as well as ethically, problematic if the autistic voice is not heard in relation to social scientific research seeking to further develop knowledge of autism’ (Chown et al., 2017). There is an epistemological lacuna with autism research that fails to reflect the lived experience of autistic individuals with their insider knowledge. Milton and Bracher stress that equal participation of autistic people will enhance research through increasing its epistemological integrity (Milton & Bracher, 2013). Autistic academics are increasingly involved in autism research, but most autism research is still undertaken by non-autistic academics. The stigma still attached to autism means that many autistic researchers do not disclose for fear of damage to their careers. For autistic academics to contribute, and to be seen to contribute, to autism research they must be enabled to disclose that they are autistic. All members of this project team are autistic. With the researchers and survey respondents all being autistic, this is a rare example of a fully autistic project.

## Results

One thousand two hundred and forty eight autistic adults responded to the surveys. The majority lived in the United Kingdom (*n* = 571), followed by the United States (*n* = 226) and Ireland (*n* = 206). Two hundred and thirteen identified as male, 806 as female and 223 as non-binary. Free comments provided 57,668 words of qualitative data for analysis. Our constructed themes are outlined in Table 2. An overarching meta-theme was also identified – a sense of epistemic injustice. This intangible permeated all other themes. Furthermore, it was particularly integral to our subsequent model. As such, it will be reported at the end of our results.

**Table 2.** Themes.

Themes	Subthemes
Early barriers	Predictability; environmental challenges; sensory challenges; interoceptive differences.
Communication mismatch	Communication differences; masking; non-manipulation
Doubt – in oneself and from doctors	Self-doubt; the unknown unknown; doctor–patient relationships.
Helplessness and fear	Learned helplessness; fearing repercussions.
Healthcare avoidance and adverse health outcomes	Healthcare avoidance; adverse outcomes; barriers have consequences.

### Theme 1: early barriers

Our respondents experienced a wide variety of early barriers to accessing healthcare. In the first instance, some respondents experienced specific challenges interpreting their internal bodily sensations to be able to decide if medical attention was needed. In particular, differences in pain thresholds/interpretation were frequently reported.

*I had appendicitis . . . because I have a high pain threshold I didn’t notice something was wrong until [after] my appendix had ruptured at least 24 hours beforehand.*

*I have an unusually high tolerance for pain, so I often am much sicker than I realize, and I don’t convey this to the GP.*

*There was a loose splinter of a bone and it was painful but I could not express my pain in a ‘normal’ way so nobody took it seriously. I wanted to visit a private doctor to get a second opinion but didn’t have enough money.*

Once certain that an appointment was needed, many outlined challenges with contacting healthcare services to make one. Foremost, significant challenges were experienced with making phone calls.

*Unable to visit my doctor due to the telephone appointment system.*

*It is a constant battle to get them to let me use written communication only and to stop them making me use the phone.*

Once an appointment was booked, respondents outlined the additional struggles with attending, including a lack of predictability, not knowing what to expect and sensory overstimulation. For example, some reported the significant barrier of needing to use public transportation to get to their doctor.

*Dependent upon public transit, which is a sensory nightmare.*

*I cannot travel by bus even when well and cannot tolerate the anxiety of waiting especially in a waiting room.*

These barriers extended into the waiting room environment, which was particularly triggering for many.

*Often I’m so distressed after waiting so long in the waiting room – especially because the appointment time advertised is rarely the time I actually go in for consultation. This confusion and unknowns means I am often very close to meltdown by the time I get in for the consultation.*

*I feel so overwhelmed from the process of visiting the doctor (being around people/sensory overstimulation in the waiting room [and] anxiety/communication difficulties) that I just have to flee outside and can’t face spending any more time in the doctors or talking to the receptionist. Sometimes I feel like if I don’t get out I will just burst into tears in the reception area.*

### Theme 2: communication mismatch

Differences in communication – including both styles and form – were experienced as pervasive issues throughout various aspects of healthcare. Some reported mishaps, anxiety and mistreatment in relation to communication with receptionists.

*I struggle with idioms. I was once told to sit tight for a referral letter and said where do you want me to sit, here or in the waiting room. Thing is I had expected the letter to be written after the appointment I was just thrown by the language. Then I felt ashamed like I had appeared demanding.*

*Receptionist brusque/confusing.*

*Rude receptionists that don’t understand neurodiversity and treat me like garbage.*

*I’ve been bullied, abused, gaslit, and mistreated by . . . receptionists at the surgery.*

Many also experienced misunderstandings when communicating with doctors and other healthcare staff.

*They think you’re purposely being rude when in reality you simply need more time to make a decision.*

*In some cases, this made it challenging to receive appropriate medical care.*

*They don’t believe my pain because I express it in words and my face and body language obviously ‘don’t match’ to them.*

Respondents, therefore, tried to mask their autism, to appear non-autistic, in the hope that they might be taken more seriously.

*Eye contact with the doctor stresses me out a lot, but I fear they won’t believe me if I’m not making eye contact.*

Respondents also acknowledged that autistic people did not know how to confidently manipulate the medical system to meet their needs in the way that non-autistic people might. In some cases, this was directly influenced by the aforementioned communication differences.

*To get [an appointment] in 1–7 days you have to . . . charm the receptionist.*

In others, this was a more explicit observation.

*It feels like trying to balance between ‘damaged enough to need help’, and ‘undamaged enough to be considered reliable’.*

Overall, there was also an awareness that being a healthcare professional themselves and understanding the medical settings and language from an insider perspective did not improve these communication mismatches.

*[I] often don’t know why I’m being so badly misinterpreted and/or misunderstood. After all I’m a nurse.*

### Theme 3: doubt – in oneself and from doctors

Respondents reported a sense of self-doubt and pre-empted guilt when considering accessing healthcare.

*I feel I am a nuisance.*

*Going to the doctor makes me feel guilty for wasting their time.*

These worries were reinforced by feelings of a lack of understanding about the possible implications of different symptoms.

*I ended up with peritonitis from a ruptured appendix because I did not recognise appendicitis symptoms as serious enough to justify making an appointment.*

These worries could be overcome through having a positive relationship with their doctor.

*A sympathetic and understanding GP means I feel less anxiety at appointments means I will be willing to go to the doctors when needed and means I attend regular screening check-ups.*

More respondents, however, experienced more negative relationships with their doctors, which fed into the healthcare barriers experienced.

*My GP hates me for misinterpreting my very precise words. I can no longer attend the doctors. I won’t go elsewhere due to change issues. I’m ill, unsupported, unmedicated and traumatised by the mistreatment at the hands of the NHS.*

*I quit seeking help for anxiety because I could not convince my doctor to accept my priorities.*

These negative relationships led to perceived questioning of their credibility as people (an epistemic injustice) and fostered a sense of frustration at their health concerns not being believed. This was felt to be due to being autistic.

*GPs treat me like a stupid child & assume I don’t understand what is being said to me because they know I have autism (I have a PhD!).*

*Doctors see my autism diagnosis and then just stop listening to me. They just say it’s stress or part of my autism.*

*I want help with anxiety not a debate about whether autistic people are capable of this or that social task.*

This led to concerns of repeated diagnostic overshadowing, where physical health concerns were attributed to their autism or an assumed state of anxiety.

*I feel that medical issues would be put down to anxiety if I told them about my autism.*

*They thought my stimming was proof I was ‘anxious’ and all my complaints were psychosomatic.*

### Theme 4: helplessness and fear

Reaching out for help to find none available, compounded by the aforementioned experiences of doubt, left respondents despondent.

*The hardest thing about going to the doctor is making the big step of making yourself vulnerable in order to ask for help but often finding help does not come.*

Such experiences promoted a sense of helplessness, whereby respondents had learned that the medical system could not, or would not, be able to support their needs.

*After never being diagnosed or getting any helpful treatment, I started to feel it was a waste of time.*

*Hospital waiting lists and tokenistic mental health services make going to GP seem futile.*

*Lack of services for autism and depression means that GP has nothing to offer.*

In some cases, this was compounded by a deep-seated fear of repercussions, should they attempt to seek help from healthcare providers.

*There are no options for help here. I’ve been tormented physically assaulted told to Google a better way to suicide and had a surgeon try to restrain me to force non-essential surgery on me or they call the police or psych services and they assault you. Getting help in anyway is not safe here.*

*I am scared to say what I truly feel to my GP (whom I do trust) as I am worried about the implications of doing so.*

*I’m afraid if the GP finds out I’m autistic she’ll decide I’m a bad parent.*

### Theme 5: healthcare avoidance and adverse health outcomes

Prior negative experiences dissuaded respondents from future contact with healthcare providers.

*I feel so disrespected by healthcare professionals that I’d rather suffer at home than set myself up for ridicule.*

In some cases, this led to a complete inability to access healthcare.

*I can no longer access healthcare so have been dealing with medical and dental issues without access to medical help for about 15 years now.*

*It’s unlikely I will ever return to A&E unless I’m unconscious and someone else takes me against my will.*

In some cases, respondents associated this total lack of access with future consequences.

*I’m gonna die one day because I didn’t go to the doctor because it was so bloody frightening.*

More broadly, respondents experienced a vast array of serious and potentially life-threatening medical outcomes. Here, the stark medical consequences speak for themselves without abstracting to our own interpretive narrative.

*Due to my difficulty describing my pain and symptoms it led to an undiagnosed . . . tumour nearly crushing my internal organs from its sheer size and needed emergency surgery which removed a melon sized tumor and an ovary.*

*Heart attack . . . preferred to sit out the chest pain even though it was agonisingly very painful and might have meant death – despite having a known heart condition and previous heart attack . . . the visit to my doctor would be too much more anxiety than could cope with.*

*I had ovarian torsion and a ruptured ovarian cyst and was in agony and was not believed by ambulance service*

*I have rheumatoid disease. I saw a Dr. at my college clinic shortly after onset of symptoms but was treated like she didn’t believe me . . . [It] has left me with irreversible joint damage.*

*I got hit by a car and didn’t go to the doctor.*

*I had an ectopic pregnancy and the fallopian tube burst. I was afraid of going to the doctor . . . When I finally went I got transferred to the ER for emergency surgery and they called my next of kin to say I might not come out of the surgery.*

*I have aortic stenosis and have recently had chest pains. I am afraid . . . afraid to schedule the appt . . . I haven’t seen a cardiologist in years.*

Some acknowledged the role of healthcare barriers in leading to these outcomes.

*It was life threatening and nearly killed me because I was too ill and too disabled to access doctors*

### How experiences may lead to barriers – an explanatory model (Figure 1)

**Figure 1.**
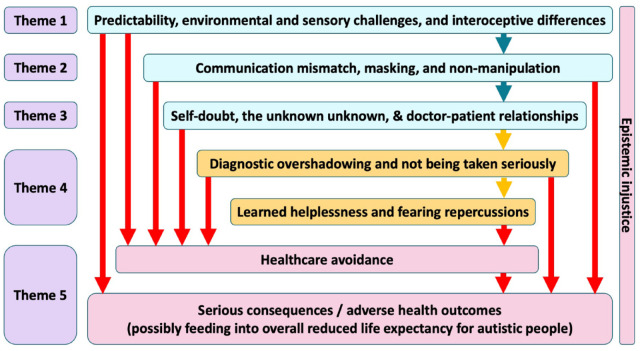
Our proposed model for explaining the reported barriers to accessing healthcare for autistic adults.

Predictability and routine are important for autistic people. However, seeking healthcare proves to be a highly unpredictable social task. It is necessary to battle busy, chaotic environments, filled with overwhelming sensory overstimulation. This may be compounded by similar environments enroute to the venue – public transport, for example.

Autistic people are known to experience interoceptive differences (Shaw et al., 2022). For example, many of our respondents reported pain awareness that differed to that which might be expected in non-autistic people – ‘*because I have a high pain threshold I didn’t notice something was wrong until my appendix had ruptured at least 24 hours beforehand*’. These differences, in combination with the lack of predictability and the environmental/sensory challenges presented by medical settings, can create a greater communication mismatch between autistic people and doctors. Our respondents reported feeling the need to mask their autism to lessen this mismatch. However, it is well documented that masking is associated with poor mental health outcomes in autistic people (Mandy, 2019). The interplay of the communication mismatch and actively focusing attention on masking can be highly stressful. Autistic people tend to interpret rules and instructions literally (Vicente & Falkum, 2023) and may be unaware of the ways in which others might be able to manipulate the healthcare system to access appropriate care. Autistic people attempting to access healthcare may therefore be competing for scarce resources such as appointments with non-autistic people who have an advantage. This was reflected by our respondents. The nature of the doctor–patient relationship therefore becomes key in guiding the outcome of consultations. A positive relationship seemed to lead to increased satisfaction and perceived improved outcomes. A negative doctor–patient relationship, however, resulted in feelings of not being taken seriously and experiences of diagnostic overshadowing – ‘*they thought my stimming was proof I was* “*anxious*” *and all my complaints were psychosomatic*’. Over time, this led to learned helpless states, where respondents had developed feelings that the healthcare system was not able to offer them any support. Combining this learned helplessness with fears of repercussions from medical contact seemed to lead to healthcare avoidance – ‘*I feel so disrespected by healthcare professionals that I’d rather suffer at home than set myself up for ridicule*’. Finally, this healthcare avoidance seemed to feed directly into the serious, adverse health outcomes reported by our respondents. As outlined in Figure 1, various stages also seemed to feed directly into healthcare avoidance and/or serious consequences.

### Meta-theme: a sense of epistemic injustice

Permeating our entire thematic analysis, and underlying the entire process seen in Figure 1, was a sense of epistemic injustice. In this context, it became clear the respondents felt reduced to a single-dimensional label of autism, and therefore their own thoughts, intelligence and self-awareness were subconsciously considered less valuable or less credible by doctors – ‘*GPs treat me like a stupid child & assume I don’t understand what is being said to me because they know I have autism (I have a PhD!)*’.

## Discussion

Our findings give voice to the healthcare experiences of autistic people. Following analysis, their stories outline a journey through which healthcare access barriers may lead to adverse health outcomes for autistic people (Figure 1). The barriers identified were broadly in line with previous studies and centred around patient–provider communication challenges and sensory issues. Adverse outcomes, while self-reported, were in many cases very clearly medically serious – thus demonstrating a plausible association between the subjective difficulties accessing healthcare and the objective healthcare outcomes and premature mortality experienced by autistic people.

### A triple empathy problem

Communication and empathic understanding with and from respondents’ doctors seemed to play an important role in guiding the self-reported outcomes in our study. A ‘key’ aspect of psychological models of human social interaction is that people have an innate ability, and/or develop the ability, to understand the mental states of others. This is called theory of mind (Carlson et al., 2013). The literature on autism is replete with scholars arguing that autistic people have difficulty understanding other minds. There are even examples of scholars who consider that autistic people are unable to empathise at all (Barnbaum, 2008). Milton argued that the only difficulty autistic people have with empathy and communication is when interacting with non-autistic people and that non-autistic people experience similar difficulty interacting with autistic people (Milton, 2012). He called this the double empathy problem (also known as cross-neurological theory of mind). Autistic people often develop a greater understanding of non-autistic social interaction than vice versa (Milton, 2012, 2017). This is because autistic people have no choice but to develop such an understanding to survive in a non-autistic world, whereas non-autistic people do not need to understand autistics. Several studies have produced evidence in support of double empathy/cross-neurological theory of mind (Crompton et al., 2020; Heasman & Gillespie, 2019; Mitchell et al., 2021).

During our analysis, it became clear that there was a perceived mismatch in communication, understanding, agendas and empathy between respondents and their doctors, which appeared more complicated than, and not fully explained by, the double empathy problem. Many of our respondents referred to matters that demonstrated bi-directional difficulties between themselves and their doctors – some of which we explored within Theme 2 (‘communication mismatch’). Autistic people are potentially less likely to infer the meaning of unspoken messages from non-autistic doctors. For example, concern on the part of doctors may often be conveyed non-verbally, so this message may be missed or misinterpreted as nonchalance or even contempt. However, this two-way mismatch did not feel fully representative of the social phenomenon we were witnessing within our data. As such, in relation to healthcare access for autistic adults, we propose that a **triple empathy problem** was at play.

Considering healthcare delivery more widely, it is common for bi-directional communication difficulties to exist between doctors and patients. It is well known that mismatched communication, understanding and agendas within medical consultations, alongside poor doctor–patient relationships, form barriers to clinical care for all patients, whether autistic or not – leading to reduced standards of care and worse clinical outcomes in the short term (Hinchey & Jackson, 2011; Mamede et al., 2017; Zhang et al., 2020). This likely stems from the fact that medicine has its own culture, language and practices. Those working within medicine are trained in and spend years experientially learning to join and embody this culture, which would naturally be alien to those external to it. For example, the primary role of a GP is often to rule out serious causes for symptoms, and then to explore common ones (Foot et al., 2010). Similarly, from an insider perspective on medicine, it is all too common for symptoms to go unexplained beyond this, even following investigation from specialist services. From a non-medical perspective, however, the concept of medically unexplained symptoms, or the lack of explanation, can be highly distressing (Kirmayer et al., 2004). This can foster tension between GPs and patients in such contexts, be they autistic or not.

**Figure 2.**
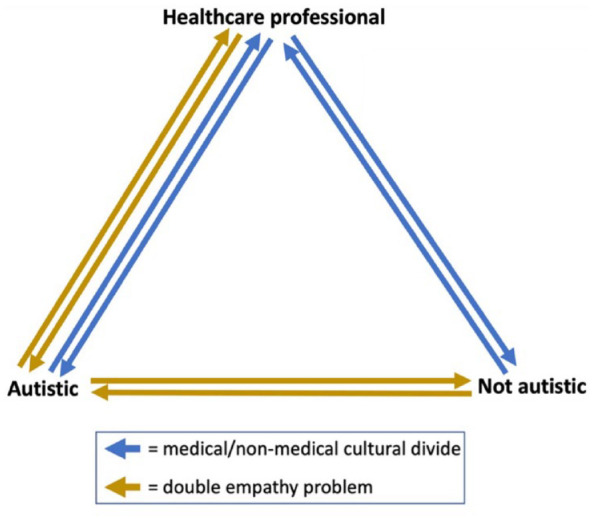
The triple empathy problem.

The cumulative toll of these cultural and agenda differences, which occur between all patients and their doctors, concurrent to the double empathy problem, seemed to have a particularly strong impact on healthcare experiences and perceived outcomes for our autistic respondents. When autistic respondents described interactions with non-autistic healthcare providers (undoubtedly the majority), this dynamic took on a three-dimensional quality. Therein lies our triple empathy problem (Figure 2). Patients struggle to see their doctor’s perspective, and doctors can also struggle to see their patients’ perspectives. For example, when doctors are patients themselves, they experience healthcare with their own medical knowledge. The difficulty is seeing the perspective of a patient without any medical knowledge. Similarly, autistic people struggle to see non-autistic people’s perspectives and vice versa. So, it proves even harder for autistic patients to see their (non-autistic) doctor’s perspective, and even harder for (non-autistic) doctors to see autistic patients’ perspectives. This extension of, and addition to, the double empathy problem is further supported by our finding that respondents with healthcare backgrounds did not report better experiences within healthcare access.

This triple empathy problem may also be at play when autistic people interact with other professions and services, such as education, social care or the justice system. Future studies might interrogate this dynamic to explore whether autistic people are exposed to systemic disadvantage within other sectors.

### Healthcare access and adjustments

While minority stress theory can be used to provide a social *explanation* for the poorer health experienced by autistic people (Botha & Frost, 2020), and thus the excess mortality experienced (Hirvikoski et al., 2016), preventive solutions will require substantial shifts in societal and cultural practices. In the interim, access to healthcare for autistic people is a key consideration both at system-wide and individual practice level. It is imperative that changes be made in medical practice to facilitate good care. Figure 1 highlights some potential areas (the areas in blue, for example) where we might target interventions/changes to improve healthcare access experiences and outcomes for autistic people.

**Figure 3.**
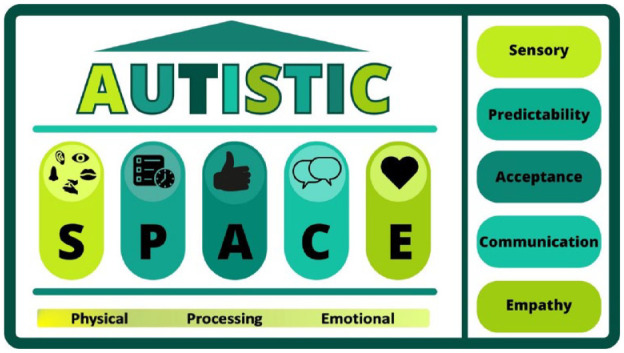
Autistic SPACE framework. Reproduced from Doherty et al. (2023).

We have used insights gained during this project to develop a simple, memorable framework to enable clinicians to meet the needs of autistic people in healthcare settings. ‘Autistic SPACE: a novel framework for meeting the needs of autistic people in healthcare settings’ (Doherty et al., 2023) outlines in detail our suggested accommodations which clinicians can easily adopt for autistic patients in all healthcare settings including general practice. The infographic reproduced here (Figure 3) incorporates the most common aspects of autistic needs in an easily memorable manner. The acronym ‘SPACE’ stands for Sensory needs, Predictability, Acceptance, Communication and Empathy – and three further domains of autistic experience where the ‘SPACE’ principles applied are represented by physical space, processing space and emotional space (Doherty et al., 2023).

Any practical adjustments must also be accompanied by education of healthcare professionals about the specific needs of autistic patients, the excess morbidity and mortality associated with not meeting these needs and the ways in which the practice changes can benefit autistic patients. This could be done during training, during continued professional development and as practices change their systems to accommodate these needs.

Further, and of equal importance, systems changes and the reasons they have been implemented should be publicised to all patients so that they know what is available and will be encouraged to initiate any needed contact with healthcare resources. In addition, the recruitment of autistic physicians and healthcare professionals could ultimately help to overcome the triple empathy problem that we have described, through the intuitive understanding that comes from their dual insider positioning within the autistic population and within the healthcare system. While many barriers to medical training have been reported by medical students (Shaw, Doherty, & Anderson, 2023), autistic doctors do indeed report a wide variety of strengths that they bring to their clinical work (McCowan et al., 2022) – including one study finding that 73% of autistic doctors felt that being autistic was helpful in their clinical work (Shaw, Fossi, et al., 2023).

### Strengths and limitations

As with any study, there are a variety of strengths and limitations to our approach that are important to consider. The findings we report here are qualitative in nature. These results are not assessed for generalisability. However, our philosophical and methodological underpinnings do not seek or claim generalisability. Instead, our findings provide insight into the world of autistic adults trying to access healthcare, advancing knowledge and giving voice to their emotional stories – an epistemological advantage in itself (Anderson, 2006). It is worth acknowledging here that this study does not include the perspectives of healthcare staff, so only sheds light on one side of these stories. Future studies might benefit from considering the experiences of healthcare staff in relation to treating autistic patients.

Our study also benefits from an insider approach. We are all autistic adults ourselves. Furthermore, six of us are also medical doctors. These insider perspectives helped to provide important insights into the study design and the interpretation of our findings.

It is also important to consider our data collection method as both a potential strength and limitation. Due to its asynchronous, online nature, it is likely that the online survey approach is a more accessible data collection tool for autistic participants. This is an important strength. The resulting very large number of participants also allowed us to identify clear trends in the experiences and stories reported on a larger scale. However, it also leads to the loss of the individual voices and narrow situational context that a smaller sample size provides.

## Conclusion

Autistic people report barriers to accessing healthcare. This may well influence our reduced life expectancy and higher rates of co-occurring conditions. Here, our results have given voice to the stories of autistic adults in trying to access healthcare. We have used this exploratory data to build a model, which may offer some insight into the relationship between barriers to access and poor outcomes. While this model is grounded in qualitative data, it opens the avenue to further research in the future – for example, through quantitative study testing such associations. Also grounded in our findings, we have built upon the double empathy problem, suggesting a triple empathy problem may be at play in relation to communication between autistic people and non-autistic doctors in medical settings. This may benefit from the inclusion of more autistic doctors in the medical workforce. This is another area in need of further study. Following our findings, we have also made recommendations for best practice when providing healthcare services for autistic people, such as general practice, and have highlighted the need for autism training in medical curricula.
